# Advances in Intracorneal Ring Segment (ICRS) Implantation for Keratoconus: A Comprehensive Literature Review, Clinical Insights, and Future Prospects

**DOI:** 10.3390/jcm14134454

**Published:** 2025-06-23

**Authors:** Pablo Morales, Juan A. Durán

**Affiliations:** Instituto Clínico Quirúrgico de Oftalmología (ICQO), 48010 Bilbao, Spain; pmoraleslpz@gmail.com

**Keywords:** keratoconus, ring segments, ectasia, corneal transplantation, corneal allogenic intrastromal ring segments

## Abstract

Keratoconus is a progressive corneal disorder that causes thinning and irregular astigmatism, often leading to significant visual impairment. In the advanced stages, surgical interventions are necessary to restore corneal shape, improve vision, and enhance contact lens tolerance. Intracorneal ring segments (ICRSs) have emerged as a well-established, minimally invasive option that not only improves vision but also has the potential to delay or prevent the need for corneal transplantation in advanced cases. Recent advancements in the ICRS implantation techniques, patient selection, and femtosecond laser technology have significantly improved the precision and safety of these procedures, reducing complications. The ability to customize the ring parameters—such as thickness, arc length, and positioning—enables a more individualized approach, particularly for patients with irregular astigmatism. Artificial intelligence (AI) is also emerging as a promising tool for optimizing ICRS planning and improving patient outcomes. Although still in the early stages, AI algorithms may refine the treatment strategies by analyzing large datasets, improving the patient selection, and predicting long-term outcomes. Corneal Allogenic Intrastromal Ring Segments (CAIRSs) offer a novel alternative to synthetic ICRSs, with advantages like improved biocompatibility and reduced extrusion risk. However, CAIRSs remain an evolving technique that requires further refinement and long-term evaluation to determine the tissue integration, the durability of the refractive outcomes, and the potential for late-onset complications. In conclusion, ICRSs continue to be a safe and effective option for managing advanced keratoconus. Ongoing refinement of the surgical approaches—combined with advancements such as femtosecond laser technology and the integration of AI—will ensure that both ICRSs and CAIRSs remain key components in the therapeutic arsenal for keratoconus, offering sustained visual improvements and the potential to delay or avoid corneal transplantation.

## 1. Introduction

Keratoconus is a progressive, ectatic corneal disorder characterized by corneal thinning and irregular astigmatism, which often leads to significant visual impairment. In the early stages, the conventional management includes spectacles and contact lenses. However, advanced keratoconus often requires surgical intervention to improve corneal shape, vision, and contact lens tolerance. Intracorneal ring segments (ICRSs) have emerged as a minimally invasive and reversible option for improving vision and potentially delaying the need for corneal transplantation. Over the past two decades, advancements in the ICRS implantation techniques, patient selection, and nomograms have significantly improved the outcomes [1]. The use of femtosecond laser technology for precise and controlled ring placement has enhanced safety, reducing the risk of complications and ensuring more predictable results [1]. Moreover, the ability to customize the ring thickness, arc length, and positioning allows for more precise corneal reshaping, particularly in cases of highly irregular astigmatism [2,3,4].

Artificial intelligence (AI) is also emerging as a promising tool in ICRS planning and development. While not yet widely implemented in clinical practice, AI-driven algorithms have the potential to refine the patient selection, improve outcome predictions, and optimize the ring segment design by analyzing large datasets, paving the way for more personalized treatment strategies in the future [5,6]. Furthermore, Corneal Allogenic Intrastromal Ring Segments (CAIRSs) have recently gained attention as a biological alternative to synthetic ICRSs. CAIRSs, which utilize allogenic corneal tissue, offer potential advantages such as better biocompatibility and a reduced risk of extrusion, expanding the range of therapeutic options for keratoconus management [1,7].

In this review, we explore the latest developments in ICRS implantation for advanced keratoconus—focusing on novel surgical approaches, technological innovations, and long-term efficacy—while sharing insights from our personal experience in clinical practice. Through our observations and patient outcomes, we aim to offer a practical perspective on the real-world application of ICRSs in the management of advanced keratoconus.

## 2. Long-Arc ICRSs for Advanced Central Keratoconus Treatment

For central ectasias (nipple/bowtie-type), based on the morphologic keratoconus classification described by Alfonso et al. [8], significantly better results have been observed with symmetrical ring implantation [9]. In eyes with mild to moderate keratoconus, short-arc ICRS implantation effectively flattens and regularizes the corneal shape, improving visual quality [1,10,11,12]. However, its effectiveness diminishes as the disease progresses. To address this, longer ICRSs of 210°, 300°, 320°, 340°, and 355° have been introduced for moderate to advanced central keratoconus [12,13,14,15,16,17,18,19,20,21].

**Figure 1:**
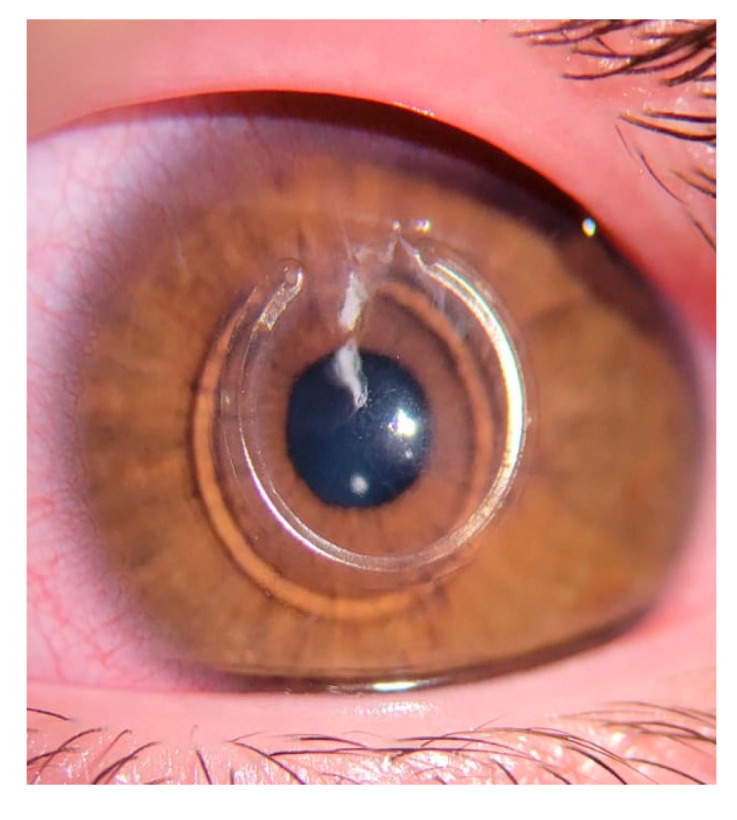
A 320° intracorneal ring segment. A 20° gap from the tip to the incision is observed, thus reducing the risk of extrusion and melting.

While longer segments have proven effective, the larger 340° and 355° rings are associated with a higher risk of complications, such as ring extrusion, corneal melting, and neovascularization [18,19,20]. These complications are primarily due to the proximity of the tip of the ICRS to the surgical incision [18,19,20]. To reduce these risks, variations in the arc length, such as 300° [12] and 320° [14,15,16,21], have been introduced (Figure 1). These options offer significant improvements with fewer adverse effects compared to those with the 340° and 355° rings [12,13,15,16,17,18,19,20,21].

The 320°- [15,16,17] and 300°-arc [12] ICRSs have demonstrated significant visual improvements with fewer complications compared to those with 340° and 355° rings [18,19,20]. A study involving the 340° ICRS found that 22.2% of eyes experienced a loss of lines in corrected distance visual acuity (CDVA), prompting the authors to recommend against its use in severe cases [18]. It is important to note that this study included both paracentral and central keratoconus, which may have influenced the outcomes. On the other hand, studies on 300° and 320° ICRSs have shown no complications when femtosecond-laser-assisted implantation was used, suggesting that the use of femtosecond lasers significantly enhances the safety of the procedure [12,15,16,17].

The 300° and 320° ICRSs also outperform the 210° ICRS and two 160° segments in terms of their safety and efficacy [13,14]. A study on the 300° ICRS, involving 42 eyes with femtosecond-laser-assisted implantation, showed significant improvements in CDVA, particularly in eyes with a preoperative CDVA worse than 0.3 logMAR (20/40 Snellen). A large portion of the participants’ eyes achieved a mean keratometry of ≤53.00 D, with a significant percentage reaching ≤50.00 D and ≤48.00 D. Since Kmean values above 53.00–55.00 D increase the risk of corneal transplantation, these findings suggest that the procedure may reduce the need for transplantation in a substantial number of cases [12].

The largest study on 320° ICRS implantation, involving 138 eyes, reported significant postoperative improvements in both Uncorrected Distant Visual Acuity (UDVA) and CDVA, with an average reduction in Kmean of approximately 5.00 D [15]. In corneas with Kmean values exceeding 60 D, flattening effects of more than 8 D were observed. Additionally, this study demonstrated a significant reduction in corneal hyperprolateness, reflected by a mean Q value change of approximately 0.8—from −1.1 preoperatively to −0.28 postoperatively [15]. This shift toward a more physiological corneal profile directly contributes to improved visual quality.

A growing body of literature supports the efficacy and safety of the 320° ICRS [15,16,17] over those of the 300° ICRS, making it the more extensively studied option for moderate to advanced central keratoconus. While the 300° ICRS may be easier to implant due to its smaller size, the 320° ICRS is often preferred for its stronger clinical validation.

In summary, both the 300° and 320° [12,15,16,17] rings offer comparable results to those of the 340° and 355° rings but with lower complication rates, as their reduced proximity to the incision minimizes risks such as extrusion, melting, and neovascularization [12,15,16,17,19,20]. These longer-arc ICRSs (210°, 300°, 320°, 340°, and 355°) remain essential for treating moderate to advanced central keratoconus, with careful patient selection and surgical techniques being crucial to achieving the optimal outcomes. For hyperprolate central keratoconus, a practical approach may involve implanting a 210° ICRS for mild cases, while 300° or 320° ICRSs may be preferred for moderate to advanced cases due to their proven efficacy, lower complication rates, and enhanced safety, particularly with femtosecond-laser-assisted implantation [12,15,16,17].

## 3. The 320° ICRS: Our Experience

**Figure 2:**
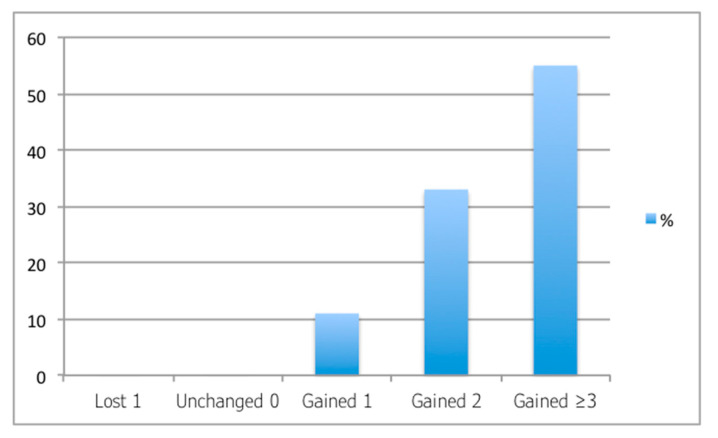
Number of lines gained/lost after implantation of 320° intracorneal ring segments.

**Figure 3:**
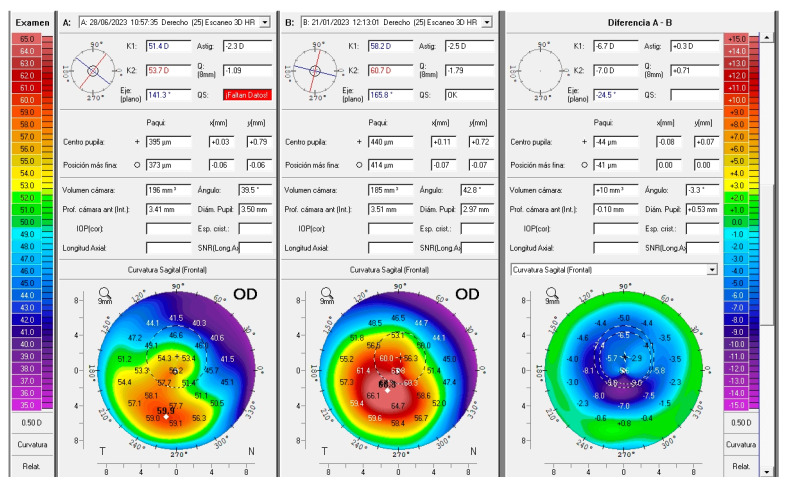
A 34-year-old female patient was referred for corneal transplantation. After undergoing implantation of 320/250 ICRS, central corneal flattening of up to 9 diopters was observed. Her uncorrected distance visual acuity (UDVA) improved from counting fingers (CF) preoperatively to 20/200 postoperatively. Her corrected distance visual acuity (CDVA) with a scleral contact lens improved from 20/50 preoperatively to 20/20 postoperatively. The Q value increased by +0.7.

In a study conducted by our group [22], we included patients with advanced central keratoconus (Kmean > 50 D) and evaluated the outcomes following 320° ICRS implantation. Our results were consistent with the previously reported outcomes, with no complications observed in any of the cases, all of which were performed using femtosecond-laser-assisted tunnel creation. Our preferred parameters for femtosecond tunnel creation were a tunnel depth of 70 to 80% of the corneal thickness at the implantation diameter, an inner tunnel diameter of 4.7 to 4.9 mm, and an outer tunnel diameter of 5.9 to 6.1 mm. More than 50% of the participants’ eyes gained at least three lines of UDVA (Figure 1), the mean Kmean decreased by approximately 5 D, and the Q value improved from −1.8 to approximately −1.0. Notably, some of the patients were referred to us as candidates for corneal transplantation (Figure 2), but with this relatively simple and reversible procedure, we were able to avoid, or at least delay, the need for keratoplasty. This is particularly significant considering that the mean age of our cohort was 28 years. Most patients achieved CDVA close to 20/20 following scleral lens fitting (Figure 3).

Based on our surgical experience—supported by several published reports—the use of femtosecond-laser-assisted tunnel creation for ICRS implantation enhances the precision, promotes the optimal segment depth, and significantly improves long-term segment retention. When performed with the appropriate surgical planning, the explantation rate remains low. Moreover, in rare cases where explantation is required, minimal adverse clinical sequelae have been observed, further reinforcing the safety profile of femtosecond-laser-assisted ICRS techniques.

In light of these findings and our clinical experience, we recommend considering 320° ICRS implantation as a relatively easy, safe, effective, and reversible option, even for severe/advanced cases of central keratoconus, whenever the corneal thickness allows. Patients should be advised that possible postoperative contact lens fitting and close follow-up are crucial and that in these severe cases corneal transplantation still remains a possible option if the results are unsatisfactory, considering this procedure as a last-resort measure in some cases.

## 4. Asymmetric-Thickness ICRSs

Asymmetric keratoconus, particularly type II (duck) and type III (snowman), is characterized by a noncoincident topographic and coma axis (>30° divergence) [8]. The standard ICRS implantation effectively corrects refractive and topographic astigmatism in these cases but does not adequately address coma-like aberrations, a primary factor contributing to poor vision [23,24]. This limitation arises because traditional constant-thickness ICRSs primarily target astigmatism without sufficient control over the primary coma.

**Figure 4:**
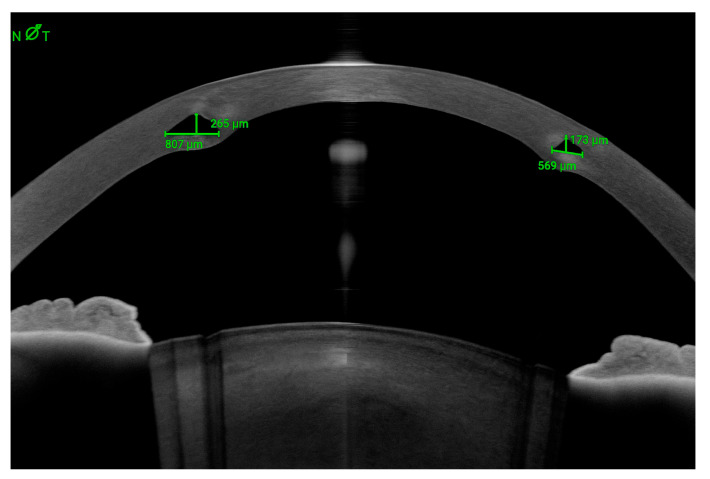
Anterior segment OCT displaying a cross-sectional image of an asymmetric ICRS, with the nasal portion of the segment appearing wider and thicker than the temporal portion.

To overcome this challenge, asymmetric-thickness ICRSs have been developed, such as the AJL**^®^** PRO+ Asymmetric ICRS (AJL Ophthalmic, Vitoria-Gasteiz, Spain) and Keraring**^®^** AS (Mediphacos Ltd., Belo Horizonte, Brazil). These segments feature variable thicknesses to address keratoconus heterogeneously, adapting to the specific phenotype (Figure 4). Multiple studies have demonstrated that asymmetric ICRSs improve refractive, keratometric, and aberrometric parameters, enhancing the visual outcomes in patients with type II and type III keratoconus [2,3,4,23,25].

A key advantage of asymmetric ICRSs is their superior control of primary coma aberrations, a major factor influencing CDVA [24,26]. Cuiña-Sardiña et al. observed a 40% reduction in primary coma aberrations at one month, with stability throughout the follow-up [23]. Similarly, Baptista et al. reported significant improvements in vertical coma aberration, the most clinically relevant high-order aberration (HOA) in keratoconus [2,27]. Despite no statistically significant reduction in the overall RMS and HOA values, patients experienced up to a five-line improvement in their UDVA, reinforcing the role of coma correction in visual enhancements [27].

Benlarbi et al. further confirmed a 38% reduction in the primary coma following AJL**^®^** PRO+ implantation, demonstrating significant refractive, keratometric, and aberrometric improvements in patients with duck-type keratoconus [4]. Additionally, their study analyzed the corneal epithelial behavior post-ICRS implantation, revealing progressive epithelial thickening from the thinnest to the thickest part of the ICRS. Notably, an increase in epithelial thickness was observed in the inner segment of the ICRS, underscoring the asymmetric impact of these implants. These changes in epithelial distribution may help compensate for stromal irregularities, potentially contributing to coma reduction [4].

In the largest series published by Prisant et al., involving 104 eyes implanted with one or two asymmetric ICRSs following manufacturer nomograms, the mean visual acuity improved from 0.82 logMAR preoperatively to 0.46 logMAR postoperatively [3]. This study concluded that asymmetric ICRSs provide a safe and effective approach to enhancing visual acuity and reducing the refractive error and mean keratometry in asymmetric keratoconus [3].

Although promising results have been observed in type II and type III keratoconus, it remains to be determined whether asymmetric-thickness ICRSs provide greater visual enhancements compared to those with symmetric ICRSs in these patients. Furthermore, despite the effectiveness of these rings for these phenotypes, it is important to consider that different implantation nomograms have been used. Standardizing the implantation protocol for these ICRSs may help optimize clinical outcomes.

## 5. ICRS Re-Implantation in a Previously Treated Cornea: A Change of Plan

**Figure 5:**
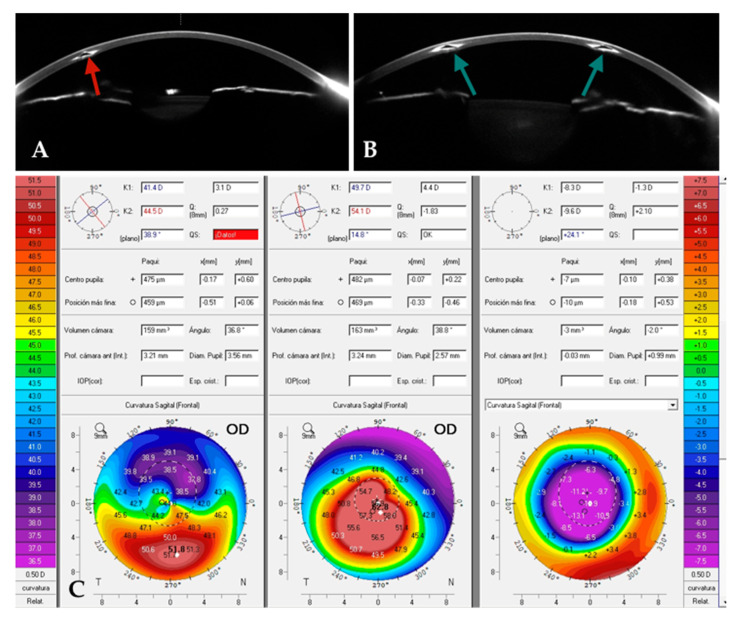
The exchange of a previously implanted single ICRS with a long-arc 320° ICRS. (**A**). Single ICRS (red arrow) preoperatively. (**B**). After exchange of single ICRS for a long-arc 320° ICRSs (green arrows) postoperatively. (**C**). A comparative preoperative axial map, showing significant flattening of up to 13 D postoperatively (C, right).

**Figure 6:**
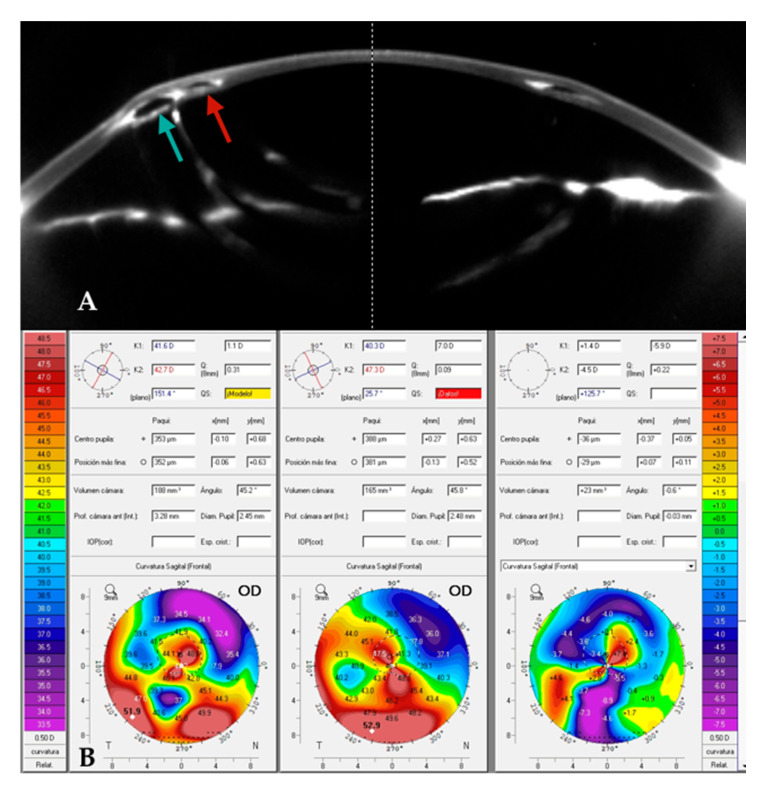
(**A**). A 320° AJL^®^ intracorneal ring segment (ICRS) (red arrow) was implanted in a patient with a pre-existing INTACS^®^ ICRS (green arrow), without requiring explantation of the latter. (**B**). Comparative axial maps: preoperative (**left**), postoperative (**center**), differential (**right**). Up to approximately 9 D of inferior flattening is observed, as is a decrease of almost 6 D in the keratometric cylinder. The patient’s CDVA improved from 0.5 to 0.8 (Decimal).

Given the absence of accurate mathematical models for reliably predicting the effects of ICRS implantation on an ectatic and biomechanically compromised cornea, anticipating the clinical outcomes remains a significant challenge [24]. The traditional nomograms primarily rely on parameters such as keratometry, the refractive error, and the cone location. However, more recent approaches have aimed to improve the precision by integrating additional data, including corneal topographic patterns, asphericity, higher-order aberrations, and the alignment of the astigmatic and comatic axes [27,28,29,30]. Despite these advancements, a consistent correlation between tomographic changes and functional visual outcomes remains elusive, as alterations in corneal morphology do not always result in predictable visual improvements. For instance, in cases where previous ICRS implants, such as INTACS^®^ (Addition Technology Inc., Lombard, IL, USA), with an optical zone (OZ) of 7 to 8 mm, have been used, creating an inner tunnel with a smaller OZ could be advantageous (Figure 5). This strategy, when combined with an assessment of the corneal phenotype, can guide the planning for a revised implantation. An alternative approach involves exchanging previously implanted ICRSs, either by reusing the existing tunnel or creating a new one—preferably using femtosecond laser technology to enhance the precision (Figure 6).

In cases of advanced central keratoconus, the use of longer-arc ICRSs, such as those with 300° or 320° arcs, may yield favorable outcomes, as previously discussed. Conversely, in cases of asymmetric keratoconus, newer asymmetric ring designs may offer better anatomical alignment and improved visual results. Given that these scenarios often involve advanced cases, where corneal transplantation may still be a consideration, it is imperative to thoroughly discuss this potential treatment option with the patient.

In this paper, we present two cases that exemplify the strategies and clinical decision-making processes involved.

Case 1: A patient with a previously implanted single ICRS (Figure 5A) was referred for corneal transplantation due to poor vision and contact lens (CL) intolerance in the right eye (OD). Corneal topography revealed a central advanced phenotype (nipple pattern), with a CDVA of 0.5 (decimal), a topographic cylinder of 5D and K2, and Kmax readings of 54 D and 63 D, respectively. Given the advanced stage of the condition, the patient was counseled on the possibility of implanting a 320° ICRS as a last-resort measure. The previously implanted ICRS was explanted, and a 320°-arc ICRS (Figure 5B) was implanted using the same tunnel (Figure 5A). Postoperatively, UDVA improved from 0.2 to 0.8 (decimal), and CDVA increased to 0.8. Significant corneal flattening was observed, with reductions in K2 and Kmax of up to 10 D and 12 D, respectively. Additionally, the Q value improved markedly, shifting from −1.8 preoperatively to approximately 0.2 postoperatively (Figure 5C).

Case 2: A patient with a previously implanted INTACS^®^ was referred due to poor vision and contact lens (CL) intolerance. Preoperative corneal topography revealed a central, relatively symmetrical bowtie pattern (Figure 6B, center). The patient was counseled on the option of implanting a new ICRS with a smaller OZ within the margins of the existing INTACS^®^. A femtosecond-laser-assisted tunnel was created within the original tunnel but at a smaller OZ (Figure 6A), and two 120° ICRSs were implanted according to the manufacturer’s nomogram. Postoperatively, the keratometric cylinder decreased by approximately 6 D, with inferior corneal flattening of approximately 9 D (Figure 6B, right). The manifest cylinder was reduced from 5 D to 2 D, and the CDVA improved from 0.5 to 0.8 (decimal).

Both cases illustrate how newer nomograms and advanced ICRS designs can enhance the visual outcomes in patients with previously implanted ICRSs who had unsatisfactory results. The improved corneal profile reduces the HOAs and enhances the asphericity (Q value), likely contributing to the observed improvements in visual acuity. However, patients should be counseled on the potential need for contact lens adaptation to optimize their vision further. Additionally, the use of a femtosecond laser in these cases provides a significantly safer and more precise approach.

## 6. Corneal Allogenic Intrastromal Ring Segments (CAIRSs)

Since their introduction by Jacobs et al. in 2018 [31], CAIRSs have been positioned as an effective, biocompatible, and safe alternative to ICRSs in managing keratoconus [7,31,32,33,34]. The CAIRS technique involves the implantation of ring segments derived from donor corneal stroma into an intrastromal tunnel at approximately 50% of the minimum pachymetry within the 7 mm optical zone.

In the initial series published by Jacobs et al., 24 eyes with progressive keratoconus (Amsler–Krumeich stages 1–4) underwent CAIRS implantation immediately followed by accelerated corneal cross-linking (A-CXL) [31]. The outcomes demonstrated sustained visual improvements over 12 to 18 months, with the mean UDVA and CDVA improving by 1 and 2 lines, respectively. The SE decreased by a mean of 4.0 D, K2 and Kmean decreased by 3.5 D, and Kmax decreased by approximately 2.5 D [31].

A recent comprehensive review of CAIRS implantation analyzed the outcomes in 389 eyes across nine studies conducted between 2018 and 2024 [7]. All cases utilized femtosecond laser technology for intrastromal tunnel creation. Ring segments were implanted at a stromal depth of 35–50%, with their inner diameters ranging from 4.0 to 6.5 mm and their outer diameters ranging from 6.8 to 8.0 mm. CDVA improved from a mean of 0.50 to 0.19 logMAR, while the SE decreased by a mean of −4.75 D. The keratometric values (K1, K2, Kmean, Kmax) decreased by an average of 4.0 to 4.5 D. As in conventional ICRS procedures, nomograms were used to guide the selection of the ring segment shape, size, thickness, and implantation location based on individual patient parameters such as keratometry and corneal thickness. In this review, two surgical nomograms were compared, with both yielding comparable visual and refractive outcomes.

In the longest follow-up study to date, CAIRS implantation demonstrated stable visual and topographic results over a three-year period [32]. This study utilized pre-shaped, sterile allograft rings (KeraNatural, VisionGift, Portland, OR, USA), sterilized via an electron beam and stored at room temperature for up to two years. These refinements in CAIRS preparation potentially enhance surgical flexibility and patient safety while reducing contamination risks [32]. UDVA improved from 0.96 to 0.41 logMAR, CDVAdecreased from 0.72 to 0.22 logMAR, and the SE decreased by 5.4 D. The keratometry values, including K1, K2, Kmean, and Kmax, decreased by an average of 3.0–3.5 D. No complications, such as melting or extrusion, were reported. In addition, the epithelial remodeling was consistent with the patterns seen with ICRSs, characterized by epithelial thinning over the ring and peripheral thickening [32].

Jacobs et al. further investigated a customized CAIRS approach, adjusting the ring design according to the corneal topography [33]. In a series of 32 eyes followed for one year, the visual and topographic outcomes were consistent with previous studies [31].

A retrospective comparative study by Asfar et al. evaluated the outcomes with CAIRSs versus those with synthetic ICRSs, specifically the INTACS^®^ SK, with follow-up periods of 9 and 10 months, respectively [34]. To ensure clinically meaningful comparisons, a score-matching strategy was applied to balancing the groups based on age, segment parameters, and key corneal metrics. The analysis demonstrated significant improvements in CDVA, refractive astigmatism, Kmax, and vertical coma in both the CAIRS and INTACS^®^ SK groups. Importantly, no major complications were reported in either cohort during the follow-up period [34].

To date, no cases of extrusion have been documented in clinical studies involving CAIRSs [7,31,32,33]. In contrast, synthetic ICRSs are known to carry a higher risk of extrusion, with reported rates ranging from 1% to 30% [10,35]. This discrepancy may be partially attributed to the biomechanical properties of the allogeneic tissue used in CAIRSs, as well as the surgical differences observed in both procedures. Specifically, superficial implantation and the placement of segments too close to the main incision site have been identified as primary contributors to extrusion with synthetic ICRSs.

Owing to their favorable complication profile, CAIRSs present additional clinical advantages over synthetic ICRSs. Notably, they can be safely implanted in thinner corneas and at shallower stromal depths owing to enhanced tissue biointegration and a lower risk of stromal melt or necrosis, as demonstrated in recent clinical studies [7,31,32]. Additionally, because the allogeneic corneal tissue closely matches the refractive index of the host cornea, CAIRS segments can be implanted using smaller OZs, potentially reducing the incidence of visual disturbances such as glare and halos [7,34].

In addition, from an immunological perspective, CAIRSs appear to carry a low risk of rejection, attributed to the immune-privileged nature of the cornea and the absence of vascularization at the site of implantation [7,31,34]. However, it is important to acknowledge that the long-term safety data remain limited, and further investigation is needed to fully assess the risk of late-onset immune responses [7].

Taken together, the findings from these studies support the role of CAIRSs as a safe and effective alternative to synthetic ICRSs for the management of keratoconus [7,31,32,33,34]. While the current data suggest that CAIRSs may present a lower risk profile—particularly with respect to complications such as melting or extrusion—their long-term efficacy and safety have not yet been fully established. The lack of standardization in the surgical techniques, including variations in the tissue preparation, segment thickness, preservation methods, and the absence of universally accepted nomograms, underscores the need for further research. Specifically, long-term studies are required to evaluate tissue integration, the durability of the refractive outcomes, and the potential for late-onset complications.

Given their biocompatibility, suitability for thinner corneas, and reduced risk of extrusion, CAIRSs represent an important advancement in corneal surgery, offering a promising option for the long-term management of keratoconus.

## 7. ICRS Implantation in the Pediatric Population

While ICRS implantation has been shown to be safe and effective in improving visual acuity and corneal topographic parameters in the pediatric population, current evidence does not support its role in halting the progression of keratoconus [36,37,38,39,40]. Therefore, the indications for ICRS implantation in children should be carefully evaluated, with particular emphasis on close and continued postoperative monitoring. Corneal cross-linking (CXL) should be strongly considered early during follow-up to enhance the long-term biomechanical stability of the cornea. Moreover, it is important to highlight that no clinical evidence is currently available regarding the safety or efficacy of CAIRSs in pediatric patients, warranting additional caution in their use within this age group.

## 8. Future Prospects on ICRSs and CAIRs for Keratoconus Management

As previously noted, one of the key challenges in ICRS implantation is the lack of accurate mathematical models capable of predicting its effects on ectatic and biomechanically compromised corneas [24]. Most of the existing nomograms are empirical in nature and do not fully capture the biomechanical impact of ICRSs, often resulting in unpredictable postoperative refractive and visual outcomes [27]. Furthermore, the relationship between topographic changes and visual improvements is not always consistent, as studies have demonstrated that tomographic enhancements do not necessarily translate into reliable functional gains. While clinical feedback remains essential to the iterative refinement of these nomograms, further research is required to optimize both the design of ICRS implants and the predictive accuracy of the nomograms guiding their use.

AI offers significant potential in the management of keratoconus through the application of machine learning techniques—such as artificial neural networks (ANNs) that process structured numerical data—and deep learning approaches, particularly convolutional neural networks (CNNs) capable of analyzing raw image data, including corneal topography and anterior segment OCT. By integrating large-scale clinical datasets—including demographic, topographic, tomographic, biomechanical, and epithelial mapping parameters—AI holds substantial promise for developing more accurate and predictive models for ICRS implantation, potentially surpassing the capabilities of traditional research methodologies. The application of AI in this context was demonstrated in a study by Fariselli et al. which evaluated the use of an ANN to guide ICRS implantation [5]. In this study, 40 patients were divided into two groups: one group underwent ICRS implantation based on the conventional nomograms, while the other received AI-guided implantation, with parameters such as the segment number, arc length, and thickness determined by the ANN. This neural network algorithm simulates the optimal combination of segments to achieve the best topographic outcome and corneal optical quality. The AI-guided group demonstrated superior visual outcomes, attributed to a reduction in HOAs compared to those in the group following the standard nomograms. These findings highlight the possibility of AI to enhance the predictability and effectiveness of ICRS procedures by refining the surgical planning, minimizing the variability associated with empirical nomograms, and providing more personalized treatment options.

AI-based neural networks operate as dynamic learning systems that continuously improve as they receive new data. As these systems are fed with increasing amounts of clinical input, their predictive accuracy and decision-making capabilities are enhanced. This adaptability is particularly valuable in optimizing the results of surgical interventions such as ICRS implantation, ultimately leading to more precise treatment planning and better outcomes for keratoconus patients.

Beyond ICRSs, we believe AI will play a pivotal role in advancing CAIRSs by enabling further customization and optimization of the treatment strategies. We are confident that AI has the potential to integrate a variety of therapeutic approaches into the management of keratoconus, including excimer laser procedures, corneal cross-linking, and phakic lens implantation. By combining these techniques through an AI-driven analysis, we anticipate the development of patient-specific treatment plans that could optimize the visual outcomes and ensure long-term corneal stability. Additionally, we strongly believe AI could be instrumental in the continuous refinement of implantation nomograms, enhancing the precision of surgical planning and improving the predictability of the clinical outcomes. All of this belief is rooted in the rapid development of AI across numerous fields, with AI already showing promising results in ICRS implantation, as demonstrated in recent studies [5,6].

## 9. Conclusions

ICRSs have been well established as an effective therapeutic option for improving the visual outcomes in patients with keratoconus. Advances in the understanding of higher-order aberrations have provided critical insights into the biomechanical effects of ICRSs, facilitating the development of more refined and efficacious designs. The introduction of long-segment and asymmetric ICRSs has yielded promising results in the management of central advanced and asymmetric keratoconus, respectively, thereby expanding the therapeutic applications of these implants. Additionally, CAIRSs have emerged as a promising option for the management of keratoconus, with growing evidence supporting their clinical applicability. However, further refinement—including standardization of the surgical techniques, tissue preparation, and segment parameters—is needed to optimize the outcomes. Long-term studies are warranted to assess the tissue integration, the stability of the refractive results, and the potential for late-onset complications.

Future advancements in keratoconus treatment are poised to benefit from the synergistic integration of ICRSs and CAIRSs with a range of other therapeutic modalities. The combined application of these implants with topography-guided treatments, corneal cross-linking, phakic intraocular lens implantation, and emerging contact lens technologies holds considerable promise in enhancing visual rehabilitation and optimizing the overall treatment efficacy. Moreover, AI is expected to play a pivotal role in refining surgical planning and nomogram development, further improving the treatment precision and predictability. These innovations collectively expand the therapeutic possibilities for keratoconus, ultimately leading to enhanced patient outcomes and a better quality of life. We envision that the integration of AI-driven models will revolutionize keratoconus management by fostering highly personalized, data-driven approaches, significantly reducing the variability and unpredictability associated with the current treatment strategies.
